# Supplementary material for: RET rearrangements are actionable alterations in breast cancer
Source: Nat Commun. 2018 Nov 16;9:4821. doi: 10.1038/s41467-018-07341-4 (PMC6240119; doi:10.1038/s41467-018-07341-4)
Supplement: Supplementary file 1 — Supplementary Information [file 41467_2018_7341_MOESM1_ESM.pdf]

SUPPLEMENTARY INFORMATION

RET rearrangements are actionable alterations in breast cancer

Paratala et al.

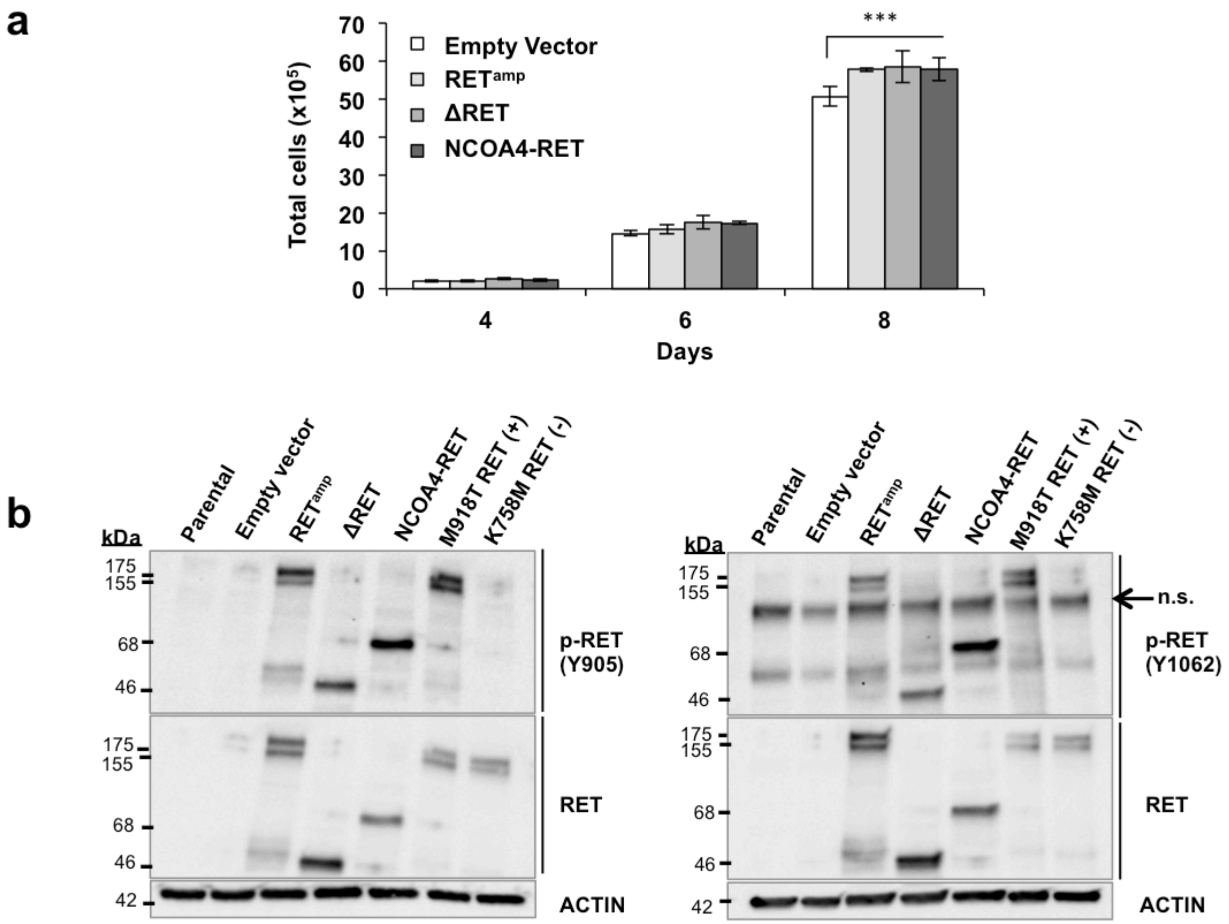

Supplementary Figure 1. Growth rates and constitutive kinase activation in MCF10A cells. **(a)** Growth rates of MCF10A cells expressing RET alterations. Cells were plated at $0.25*10^5$ cells at day 0 and counted on days 4, 6, 8. Error bars represent s.d. for n=3 experiments, ***p-value < 0.001 between vector and each of the RET altered groups at day 8 after two-way ANOVA and Tukey's multiple comparison test. **(b)** Immunoblot from MCF10A cells transiently expressing RET^{amp} (overexpressing RET wildtype) at 175, 155 kDa, ΔRET at 46kDa, or NCOA4-RET at 68kDa, reveal phosphorylation of tyrosine residue 905 (left blot) and tyrosine residue 1062 (right blot) in the absence of serum stimulation and after serum starvation overnight. MCF10A cells overexpressing constitutively kinase active (M918T) and kinase inactive (K758M) full-length RET variants serve as positive (+) and negative (-) controls respectively. n.s. represents non-specific band around 100kDa for the blot on the right. ACTIN serves as loading control.

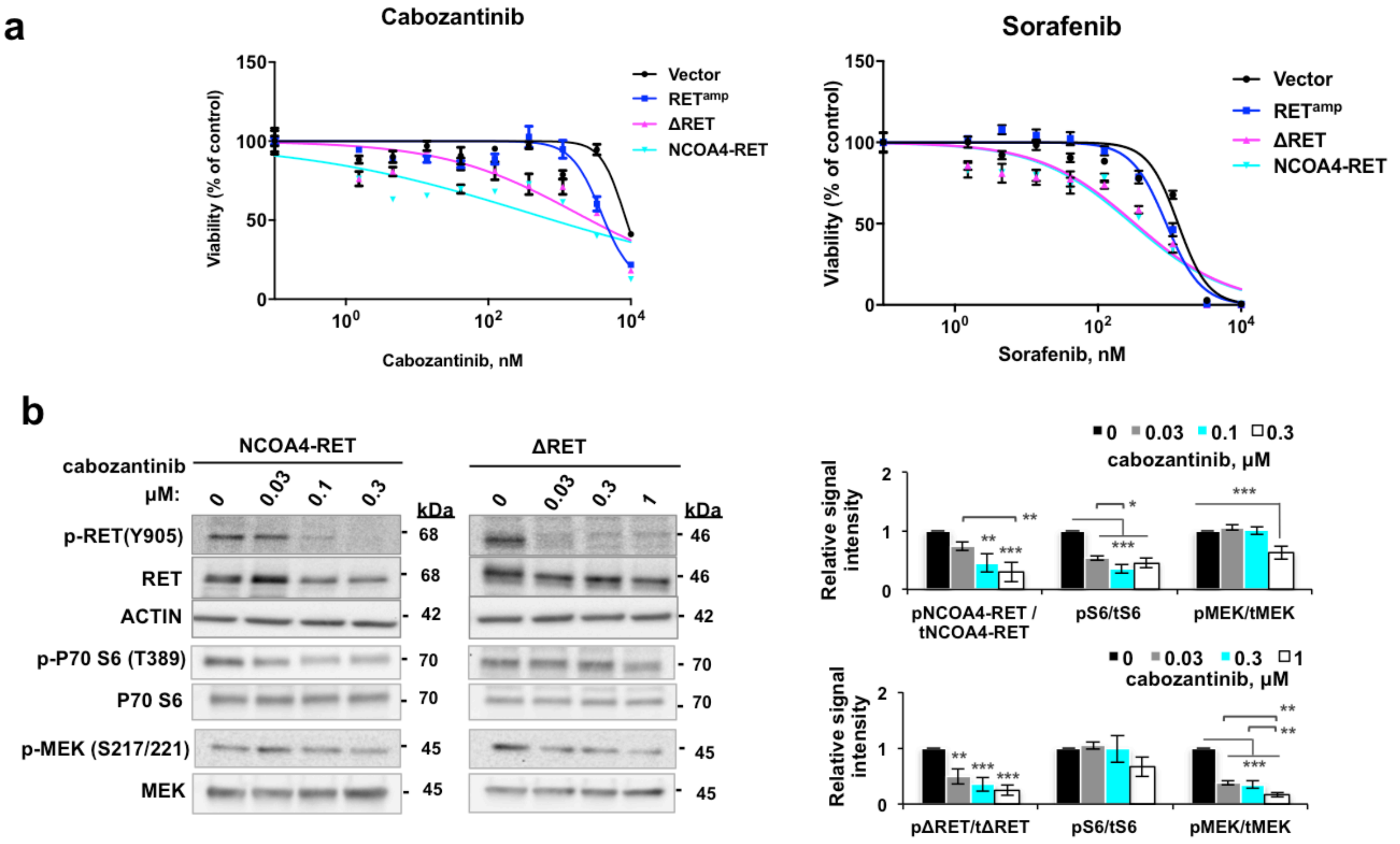

Supplementary Figure 2. Dose-dependent response to RET inhibition in MCF10A cells. (**a**) Dose-response curves after 72 hours of drug treatment with cabozantinib or sorafenib in MCF10A cells expressing RET^{amp}, ΔRET, NCOA4-RET, and vector. Cell viability normalized to vehicle (DMSO) treated cells. Error bars indicate s.d. of three replicates and are representative of three independent experiments (n=3). (**b**) Western blot indicating inhibition of RET fusion kinase, MEK and P70 S6 signaling with increasing concentration of cabozantinib in MCF10A cells transiently expressing NCOA4-RET or ΔRET. Measurements were made after overnight serum starvation and 1h of incubation with cabozantinib in the absence of serum. 0 represents vehicle DMSO treated cells. Graphs represent image densitometry analysis of western blots from three independent experiments (n=3). Ratio of phosphorylated to total proteins is measured at each concentration and mean values with error bars indicating s.d. are plotted relative to DMSO treated control. $p \leq 0.05$(*), ≤ 0.01(**) and ≤ 0.001(***) by ordinary one-way ANOVA with Tukey's multiple comparisons test. Open-ended brackets depict comparison between the indicated group and each of the groups under the bracket. Where brackets are absent, comparison is with DMSO control.

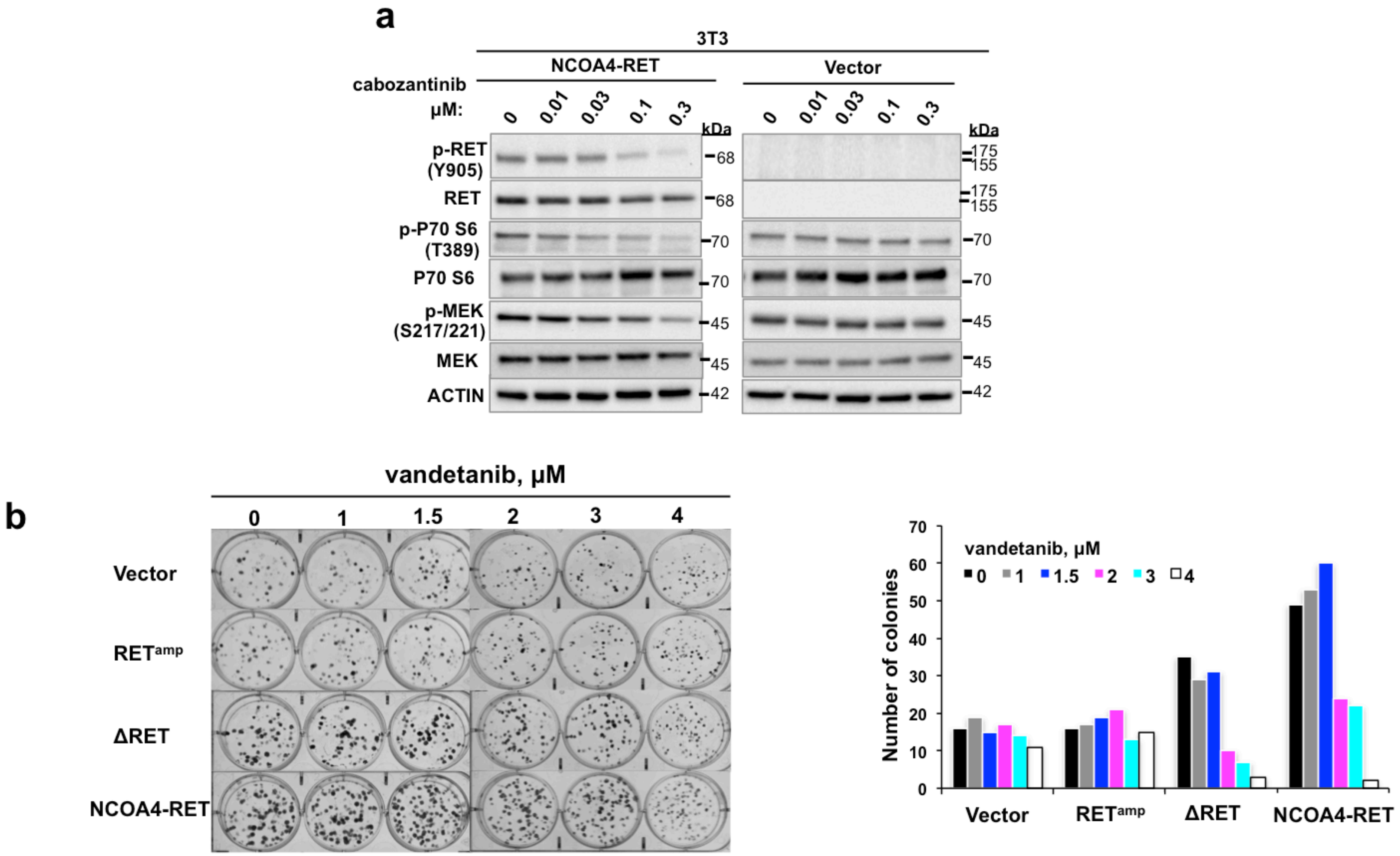

Supplementary Figure 3. Response to RET inhibitors in NIH/3T3 cells. (**a**) Immunoblot of NIH-3T3 cells transduced with NCOA4-RET or vector and dose-dependent inhibition of phosphorylation of RET kinase and downstream signaling with cabozantinib for NCOA4-RET. Measurements were made after 15 minutes of incubation with compound in the absence of serum after serum starvation overnight. (**b**) NIH/3T3 cells transduced with ΔRET and NCOA4-RET show reduction in number of colonies upon treatment with increasing concentrations of vandetanib for 10 days. Graph shows quantification of colony numbers. 0 refers to DMSO control.

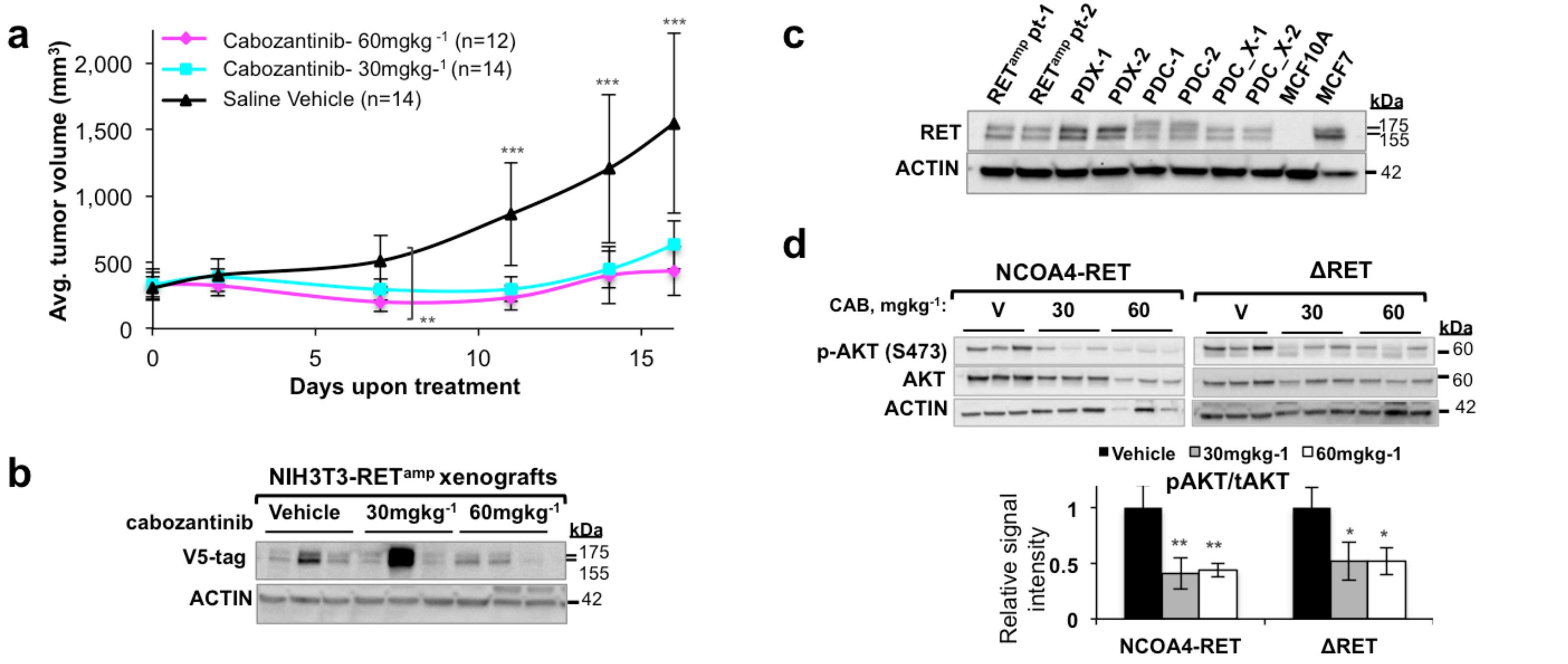

Supplementary Figure 4. Growth inhibition and protein expression in RET altered xenografts. (**a**) Mean tumor volume was measured in NIH/3T3 xenografts driven by RET^{amp}. Mice were treated with either cabozantinib at 30mgkg^{-1} (n=14), 60mgkg^{-1} (n=12) or saline vehicle (n=14) control for 16 days. ***$p<0.001$ between vehicle and treatment groups at days 11, 14, and 16. **$p<0.01$ between vehicle and 60mgkg^{-1} after two-way ANOVA with Tukey's multiple comparisons test. (**b**) Immunoblot for V5-tag measured in tumor lysates at the end of 16-day treatment in mice harboring NIH/3T3- RET^{amp} tumors. Mice were treated on the day of collection for 4 hours with saline vehicle or cabozantinib (30mgkg^{-1} or 60mgkg^{-1}). (**c**) RET protein verification in a patient tissue with RET amplification (RET^{amp} pt, * in Fig.1a), RET^{amp} patient-derived xenograft (PDX), RET^{amp} patient-derived cell line (PDC), RET^{amp} patient- cell line derived xenograft (PDC_X). (n=2, where lysates are derived from different location of the same tumor, xenograft, or different passages of cell line) MCF10A breast cells as negative control, MCF7 breast cancer cells as positive control. Methods for PDC based on Pham, K., *et al.*[4] (**d**) Immunoblot of tumor lysates collected at the end of 14-day treatment in NCOA4-RET and ΔRET xenografts reveal downstream AKT signaling inhibition. Mice were treated on the day of tumor collection for 4 hours with saline vehicle (V) or cabozantinib (CAB, 30mgkg^{-1} or 60mgkg^{-1}). n=3 per treatment group. Graphs represent image densitometry analysis of western blots. Averages of phosphorylated/total AKT levels are plotted relative to average of vehicle. $p \leq 0.05$(*), ≤ 0.01(**) after ordinary one-way ANOVA with Tukey's multiple comparisons test represent comparisons between the vehicle and treatment groups. ACTIN used as loading control in all blots. Error bars in (a) and (d) represent s.d.

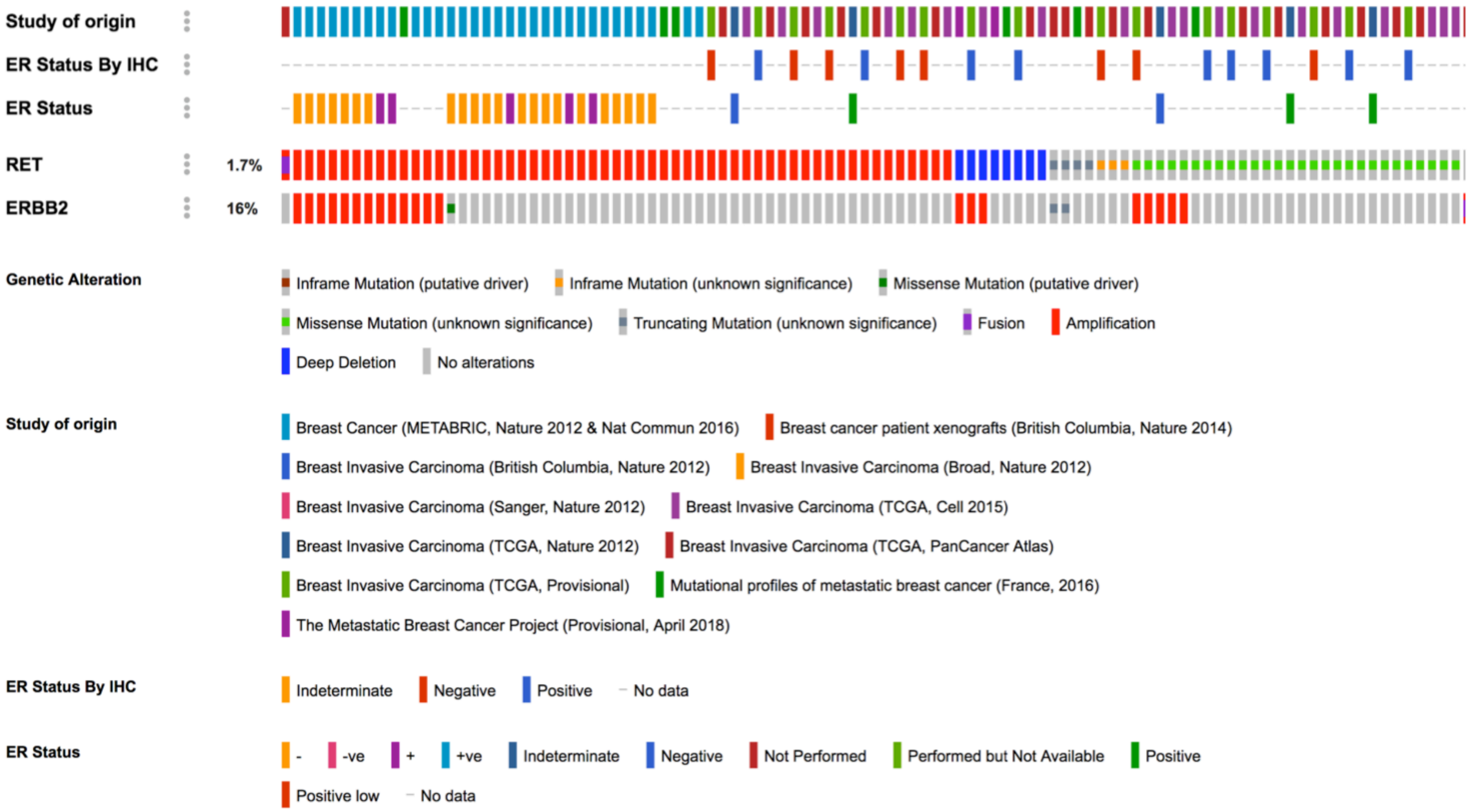

Supplementary Figure 5. *RET* altered breast cancer cases from The Cancer Genome Atlas, cBioPortal[5, 6]. 1.7% *RET* altered cases from 5,931 sequenced breast cancer cases. In cases where ER status is available, *RET* amplifications are frequently ER- (78%, p = 0.005) and *RET* mutations are frequently ER+ (70%, p = 0.02) using Fisher's exact test, two-tailed. No significant associations were observed for *ERBB2* status in *RET* altered cases.

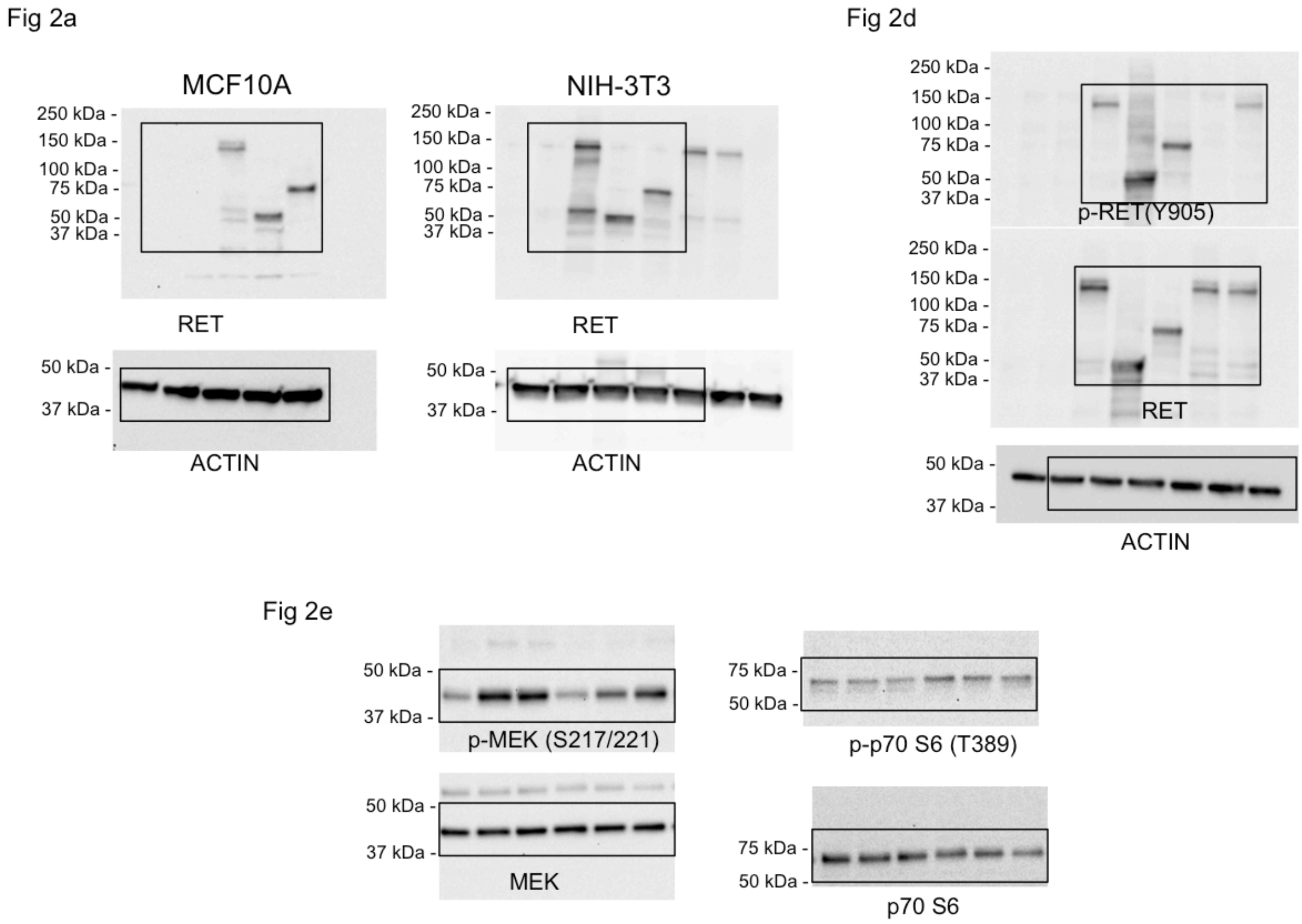

Supplementary Figure 6. Uncropped blots for Figure 2a, 2d, and 2e. Blots shown in the main article are depicted by boxed regions in each of the associated uncropped scans.

Fig 3b

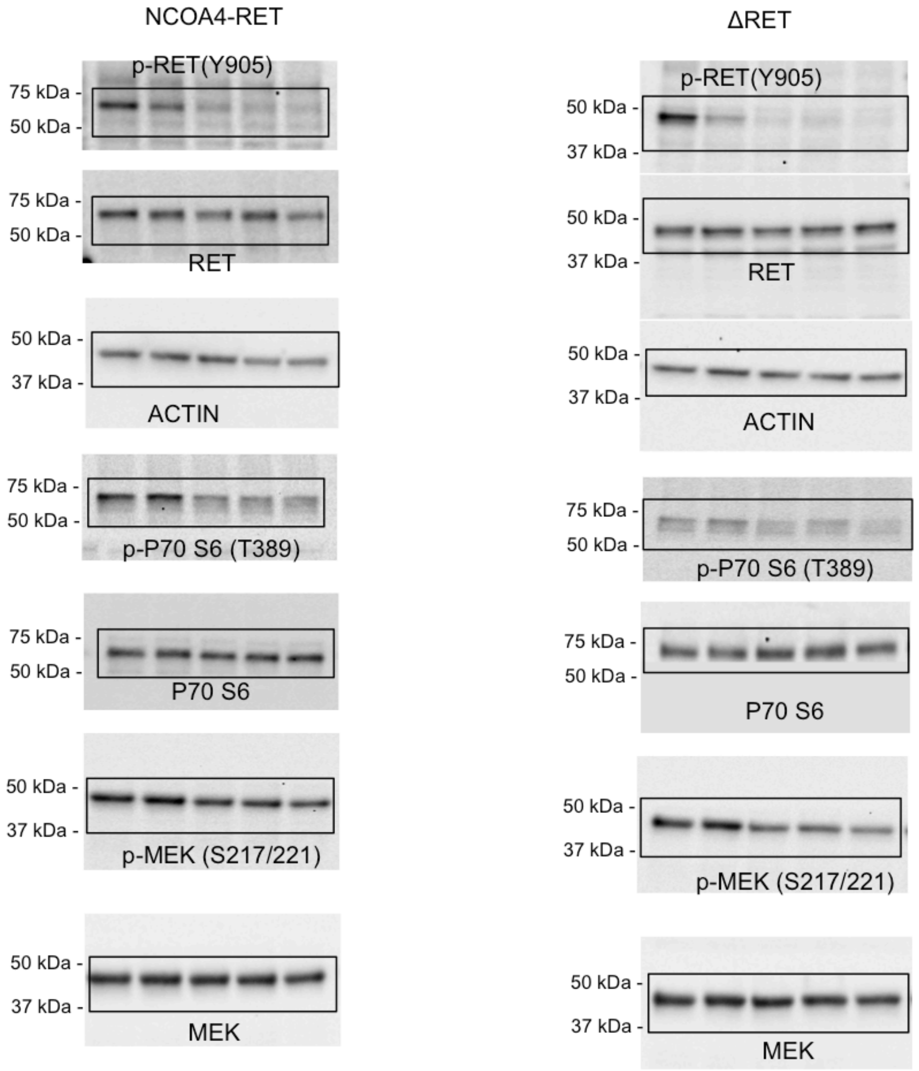

Supplementary Figure 7. Uncropped blots for Figure 3b. Blots shown in the main article are depicted by boxed regions in each of the associated uncropped scans.

Supplementary Table 1. Co-event status in *RET* altered breast cancers

Characteristics	All breast cancer cases	*RET* altered cases	*RET* activating rearrangement	*RET* uncharacterized rearrangement	*RET* missense mutation	*RET* amplification
n	9693	121	8	8	25	81
Median age, years (range)	54 (20-88)	56 (31-85)	61.5 (54-66)	60 (48-69)	52 (33-71)	54 (28-85)
Median TMB, mutations/Mb	3.6	4.5	5.2	4.5	3.6	4.5
ER-positive, n(%)	N.A	34 (34.7%)	2 (25%)	2 (25%)	15 (71.4%)	16 (25%)
ER-negative, n(%)	N.A	64 (65.3%)	5 (75%)	5 (75%)	6 (28.6%)	48 (75%)
ER-unknown, n(%)	N.A	23	1	1	4	17
HER2-positive, n(%)	1019 (10.5%)	22 (18.2%)	1 (12.5%)	1 (12.5%)	5 (20%)	15 (18.5%)
HER2-negative, n(%)	8674 (89.5%)	99 (81.8%)	7 (87.5%)	7 (87.5%)	20 (80%)	66 (81.5%)
Rank-ordered, co-occurring genomic alterations: *GENE* (%, number of altered cases)	*TP53* (56.5%, 5481)<br>*PIK3CA* (32.5%, 3155)<br>*MYC* (23.4%, 2269)<br>*11q13* (17.4%, 1688)<br>*ZNF703* (14.2% 1376)<br>*FGFR1* (13.9%, 1346)	*TP53* (80%, 97)<br>*MYC* (32%, 39)<br>*PIK3CA* (26%, 31)<br>*ERBB2* (20%, 24)<br>*MCL1* (20%, 24)<br>*PTEN* (17%, 20)	*TP53* (75%, 6)<br>*MYC* (38%, 3)<br>*11q13, ERBB2, GATA3, LYN, PIK3CA, PTEN, RB1* (25% each; 2)	*TP53* (87.5%, 7)<br>*11q13* (50%, 4)<br>*FGFR1* (37.5%, 3)<br>*MYC, MYST3, NOTCH1, PIK3CA, PIK3R1, ZNF703* (25% each, 2)	*TP53* (72%, 18)<br>*ERBB2* (24%, 6)<br>*GATA3* (24%, 6)<br>*MYC* (20%, 5)<br>*MAP2K4* (16%, 4)<br>*ZNF217* (16%, 4)	*TP53* (90%, 73)<br>*MYC* (37%, 30)<br>*PIK3CA* (30%, 24)<br>*MCL1* (26%, 21)<br>*ERBB2* (19%, 15)<br>*PTEN*(19%,15)

Italics represent genes, TMB, Tumor Mutational Burden; ER, Estrogen Receptor; HER2, Human Epidermal Growth Factor Receptor 2; N.A. not applicable

Supplementary Table 2. Germline versus Somatic variant status of RET missense mutations

Patient #	RET missense mutation	Tumor nuclei %	Allele frequency	Total gene copies	Altered copies	Status based on SGZ model*	Reported germline associations (level of risk of aggressive MTC)[1]
17	C634R	30	0.42	2	1	somatic	pheochromocytoma, hyperparathyroidism, MTC (Level C)
18	R114H	20	0.5	1	1	ambiguous_CNA model	
19	E632K	30	0.4	3	1	germline	
20	C634F	70	0.63	3	1	ambiguous_both G and S	pheochromocytoma, hyperparathyroidism, MTC (Level C)
21	V804M	40	0.51	4	2	germline	MEN 2A, MTC (Level A)
22	D925H	40	0.21	2	1	somatic	
23	V706M	30	0.59	4	2	ambiguous_neither G nor S	
24	R600Q	80	0.62	4	3	germline	MTC (Level A)
25	V591I	30	0.47	2	1	germline	
26	L633V	40	0.46	2	1	germline	
27	S462L	20	0.5	3	2	germline	
28	M918T	25	0.16	2	1	somatic	MEN2B, MTC (Level D), either S or G
29	E511K	30	0.35	3	1	ambiguous_both G and S	
30	E511K	70	0.46	2	1	germline	
31	E511K	70	0.64	3	2	germline	
32	E511K	20	0.88	2	2	germline	
33	C620F	40	0.31	2	1	ambiguous_CNA model	MEN 2A, FMTC, HSCR1
34	S462L	80	0.04	3	1	subclonal somatic	
35	V804M	70	0.52	2	1	germline	MEN 2A, MTC (Level A)
36	E511K	70	0.44	4	2	ambiguous_CNA model	
37	E232K	60	0.15	4	2	subclonal somatic	
38	C611R	60	0.13	2	1	subclonal somatic	MTC (Level B)
39	V804M	25	0.48	2	1	germline	MEN 2A, MTC (Level A)
40	T636M	20	0.03	4	2	subclonal somatic	
41	V804M	20	0.51	2	1	germline	MEN 2A, MTC (Level A)

SGZ, somatic-germline-zygosity; MTC, medullary thyroid cancer; FMTC, familial medullary thyroid cancer; CNA, copy number alteration; G, germline; S, somatic; MEN, multiple endocrine neoplasia; HSCR, hirschsprung's; * Somatic-germline-zygosity model from Sun, J.X., et al.[2] and verified from Khiabanian, H., et al.[3]

Supplementary Table 3. Gene-specific PCR Primers for RET fusions

Primer Name	Sequence
NCOA4 exon_2_Forward	ATGAATACCTTCCAAGACCAGAG
NCOA4 exon_7_Reverse	CTGACTGTTCTCCAAGGTCTGCT
RET exon 12_Forward	GAGGATCCAAAGTGGGAATTC
RET exon 19_Reverse	GAATCTAGTAAATGCATGGGAAATTC
RET exon 11_ATG_Forward	ATGACCTTCCGGAGG
RET exon 19_Reverse	GAATCTAGTAAATGCATGGG

Supplementary Table 4. List of antibodies

Antibody	Company	Catalog#	Use
phospho-RET (Y905)	Cell Signaling Technology	3221	Western Blot
phospho-RET (Y1062)	Santa Cruz Biotechnology	20252-R	Western Blot
phospho-AKT (S473)	Cell Signaling Technology	4060	Western Blot
phospho-MEK	Cell Signaling Technology	9154	Western Blot
phospho-P70 S6	Cell Signaling Technology	9234	Western Blot
RET	Cell Signaling Technology	14698	Western Blot
V5 tag	Cell Signaling Technology	13202	Western Blot
AKT	Cell Signaling Technology	9272	Western Blot
MEK	Cell Signaling Technology	9126	Western Blot
P70 S6	Cell Signaling Technology	2708	Western Blot
ACTIN	Sigma-Aldrich	clone AC-15	Western Blot
Ki-67	Spring Biosciences	M3062	Immunohistochemistry
cleaved-caspase 3 (D175)	Cell Signaling Technology	9661	Immunohistochemistry

Supplementary References

1. Marquard, J. & Eng, C. *Multiple Endocrine Neoplasia Type 2*. (2015).
2. Sun, J.X., *et al.* A computational approach to distinguish somatic vs. germline origin of genomic alterations from deep sequencing of cancer specimens without a matched normal. *PLoS Comput Biol* **14**, e1005965 (2018).
3. Khiabanian, H., *et al.* Inference of germline mutational status and evaluation of loss of heterozygosity in high-depth tumor-only sequencing data. *JCO Precis Oncol* doi: 10.1200/PO.17.00148 (2018).
4. Pham, K., *et al.* Isolation of Pancreatic Cancer Cells from a Patient-Derived Xenograft Model Allows for Practical Expansion and Preserved Heterogeneity in Culture. *Am J Pathol* **186**, 1537-1546 (2016).
5. Gao, J., *et al.* Integrative analysis of complex cancer genomics and clinical profiles using the cBioPortal. *Science signaling* **6**, pl1 (2013).
6. Cerami, E., *et al.* The cBio Cancer Genomics Portal: An Open Platform for Exploring Multidimensional Cancer Genomics Data. *Cancer discovery* **2**, 401-404 (2012).
