# Supplementary material for: RET rearrangements are actionable alterations in breast cancer
Source: Nat Commun. 2018 Nov 16;9:4821. doi: 10.1038/s41467-018-07341-4 (PMC6240119; doi:10.1038/s41467-018-07341-4)
Supplement: Supplementary file 3 — Description of Additional Supplementary Files [file 41467_2018_7341_MOESM3_ESM.pdf]

Description of Additional Supplementary Files

File Name: Supplementary Data 1
Description: List of genes in the targeted sequencing assay for exonic and intronic capture in version 1 and 2 of the FoundationOne Assay

File Name: Supplementary Data 2
Description: RET variant details, hormone receptor and ERBB2 status, site of origin and tumor purity for 121 RET altered breast cancers.
